# Clinical Evaluation of BD Veritor SARS-CoV-2 and Flu A+B Assay for Point-Of-Care System

**DOI:** 10.1128/spectrum.01807-21

**Published:** 2022-04-12

**Authors:** Katherine Christensen, Huimiao Ren, Shirley Chen, Charles K. Cooper, Stephen Young

**Affiliations:** a Becton, Dickinson and Company, BD Life Sciences-Integrated Diagnostic Solutions, Sparks, Maryland, USA; b Becton, Dickinson and Company, BD Life Sciences, San Diego, California, USA; c Tricore Reference Laboratory, Albuquerque, New Mexico, USA; University of Cincinnati

**Keywords:** BD Veritor, COVID-19, SARS-CoV-2, influenza, flu, triplex antigen assay

## Abstract

Differential diagnosis of COVID-19 and/or influenza (flu) at point of care is critical for efficient patient management and treatment of both these diseases. The study presented here characterizes the BD Veritor System for Rapid Detection of SARS-CoV-2 and Flu A+B (“Veritor SARS-CoV-2/Flu”) triplex assay. The performance for SARS-CoV-2 detection was determined using 298 specimens from patients reporting COVID-19 symptoms within 7 days from symptom onset (DSO) in comparison with the Lyra SARS-CoV-2 RT-PCR (reverse transcriptase PCR) assay (“Lyra SARS-CoV-2”) as the reference. The performance for flu A and flu B detection was determined using 75 influenza-positive and 40 influenza-negative retrospective specimens in comparison with the previously FDA-cleared BD Veritor System for Rapid Detection of Flu A+B assay (“Veritor Flu”) as the reference. The Veritor SARS-CoV-2/Flu assay met the FDA EUA acceptance criteria (86.7%; 95% confidence interval [95% CI]: 75.8 to 93.1) for SARS-CoV-2 testing compared to Lyra SARS-CoV-2. The Veritor SARS-CoV-2/Flu assay also demonstrated 100% agreement with the Veritor Flu for Flu A+B assay. For flu A detection, the lower bound of the 95% CI was 91.2%; for flu B detection, the lower bound was 90.0%. The dual detection capability of Veritor SARS-CoV-2/Flu for the etiologic agents causing COVID-19 and flu will allow efficient differentiation between the two illnesses, inform disease management, and facilitate optimal treatment.

**IMPORTANCE** COVID-19 and flu are two respiratory illnesses which share similar clinical symptoms. The BD Veritor SARS-CoV-2/Flu assay has high sensitivity and specificity for detecting the SARS-CoV-2 and influenza A/B, the two etiologic agents causing COVID-19 and flu, respectively. This dual detection capability is critical when overlap occurs between the COVID-19 pandemic and the flu season. This triplex assay will allow efficient differentiation between the two respiratory illnesses and support a point-of-care physician diagnosis to facilitate the proper treatment and disease management for patients exhibiting overlapping symptoms.

## INTRODUCTION

Coronavirus disease 2019 (COVID-19) and influenza (flu) are two potentially severe respiratory illnesses that cause morbidity and mortality worldwide. COVID-19 is the result of infection by SARS-CoV-2, which emerged at the end of 2019 (1). Since then, over 219 million COVID-19 cases and 4.55 million COVID-19-related deaths have been reported worldwide (2). The highly contagious nature of SARS-CoV-2, the lack of any natural immunity in the world’s population, and fact that there is no single, fully effective treatment for COVID-19 resulted in a global pandemic and public health crisis starting in 2020 and continuing through 2022. In the US, more than 43.7 million COVID-19 cases and over 701,000 deaths have been recorded through early October 2021 (2). Influenza is caused by influenza viruses (e.g., flu A and flu B), which occur seasonally (3). There are an estimated 1 billion cases of flu-like illness identified each year globally. Within those cases, 3 to 5 million are severe, and 29,000 to 655,000 eventually lead to flu-related deaths (4, 5). In the US, the 2019 to 2020 flu season resulted in over 38 million cases involving symptomatic illness and approximately 22,000 deaths (6).

Patients with COVID-19 often exhibit respiratory symptoms similar to those of flu, including fever, cough, fatigue, headache, and muscle aches (7). Some COVID-19 patients exhibit additional symptoms, including loss-of-smell/taste and shortness of breath; progression to severe disease can result in loss of cardiopulmonary function and death. Although several of the clinical symptoms for flu overlap with those for COVID-19, the therapeutic approaches for each illness are significantly different. The early and rapid differential detection of SARS-CoV-2 versus influenza viruses is an essential requirement in determining the proper treatment for patients with the potential for infection by each of these viruses (8). Although there were no significant flu cases between 2020 and 2021, it is anticipated that future flu seasons will return on top of the continuing COVID-19 pandemic. Accurate diagnosis of SARS-CoV-2 and/or influenza should also reduce the unnecessary burden placed on the health care system, especially during respiratory virus seasons (e.g., December to February in the US) (8).

Molecular testing, utilizing nucleic acid amplification testing (NAAT), is the standard of care for the detection of most respiratory viral infections (9, 10). However, this technology can be relatively labor-intensive and time-consuming, and laboratories generally need to have certain infrastructure and training to perform the testing (9). Rapid, point-of-care (POC) molecular testing can reduce assay times, but the cost of these assays can be prohibitive (9). In many decentralized healthcare settings, it is necessary to have a rapid testing platform that supports noninvasive specimen collection, is easy to use, and is inexpensive. Therefore, several rapid antigen tests have been developed to provide a sensitive but less expensive alternative POC assay (11, 12).

The BD Veritor System for Rapid Detection of Flu A+B (“Veritor Flu”; Becton, Dickinson and Company; BD Life Sciences-Integrated Diagnostic Solutions, Sparks, MD, USA) is FDA-cleared, and the BD Veritor System for Rapid Detection of SARS-CoV-2 (“Veritor SARS-CoV-2”; Becton, Dickinson and Company) is an FDA Emergency Use Authorization (EUA)-authorized, antigen-based testing system for use in POC settings (13, 14). The clinical performance of both tests has been demonstrated by comparison with reference PCR-based assays (15, 16). The objective of this study was to demonstrate the efficacy of a new triplex test, the BD Veritor SARS-CoV-2 and Flu A+B assay (“Veritor SARS-CoV-2/Flu”; Becton, Dickinson and Company), which allows for the simultaneous detection of SARS-CoV-2, influenza A, and influenza B viruses from one specimen.

## RESULTS

This study included 278 specimens from subjects with suspected COVID-19 within 7 days from symptom onset (DSO). The specimens were tested with the reference method for SARS-CoV-2, the Lyra SARS-CoV-2 assay. Testing with the reference method resulted in 60 SARS-CoV-2 positive and 218 SARS-CoV-2 negative specimens. The collection procedure at site D deviated from the original protocol and the integrity of that site’s specimens may have been compromised (see Materials and Methods). Although a statistically significant difference between site D and the five other sites (*P* = 0.059) was not observed when determining data poolability, results which both include and exclude data obtained by site D are reported. For all sites, Veritor SARS-CoV-2/Flu had positive percent agreement (PPA) and negative percent agreement (NPA) values of 86.7% (95% confidence interval [95% CI]: 75.8 to 93.1) and 99.5% (95% CI: 97.4 to 99.9), respectively, for SARS-CoV-2 detection compared to the reference (Table 1). Excluding site D, Veritor SARS-CoV-2/Flu had PPA and NPA values of 91.5% (95% CI: 80.1 to 96.6) and 99.5% [95% CI: 97.0 to 99.9], respectively, for SARS-CoV-2 detection. The 60 reference (Lyra PCR assay)-positive results are plotted by cycle threshold (Ct) score in Fig. S1 (in the supplemental material) divided by Veritor SARS-CoV-2/Flu-negative (*n* = 52) and -positive results (*n* = 8). The 22 to 49 years-of-age group had the highest percentage positive ratio within all positive cases compared to other age groups by both reference and Veritor SARS-CoV-2/Flu tests (Table 2).

**TABLE 1:** Performance of the Veritor SARS-CoV-2/Flu assay for detection of SARS-CoV-2 compared to that of the reference, with and without D site^*a*^

Collection site	RT-PCR reference^*b*^
D site	
PPA (95% CI)	69.2% (42.4–87.3%)
NPA (95% CI)	100.0% (89.8–100%)
Veritor (+)/Ref (+), *n*	9
Veritor (+)/Ref (–), *n*	0
Veritor (–)/Ref (+), *n*	4
Veritor (–)/Ref (–), *n*	34
Kappa coefficient	0.765
	
Other sites	
PPA (95% CI)	91.5% (80.1–96.6%)
NPA (95% CI)	99.5% (97.0–99.9%)
Veritor (+)/Ref (+), *n*	43
Veritor (+)/Ref (–), *n*	1
Veritor (–)/Ref (+), *n*	4
Veritor (–)/Ref (–), *n*	183
Kappa coefficient	0.9316
	
All sites	
PPA (95% CI)	86.7% (75.8–93.1%)
NPA (95% CI)	99.5% (97.4–99.9%)
Veritor (+)/Ref (+), *n*	52
Veritor (+)/Ref (–), *n*	1^*c*^
Veritor (–)/Ref (+), *n*	8
Veritor (–)/Ref (–), *n*	217
Kappa coefficient	0.9001

a PPA, positive percent agreement; NPA, negative percent agreement.

b Reference method was the Lyra SARS-CoV-2 RT-PCR assay.

c Analysis of the Veritor Analyzer raw data demonstrated this specimen’s results to be very close to the assay cut-off.

**TABLE 2:** SARS-CoV-2 positivity distribution by reference method or Veritor SARS-CoV-2/Flu across all age groups

Age group	*N* (%)	Reference, *n* (%)	Veritor SARS-CoV-2/flu, *n* (%)
Without D site			
18−21 yrs	8 (3.5)	5 (10.6)	4 (9.1)
22−49 yrs	127 (55.0)	26 (55.3)	23 (52.3)
50−59 yrs	53 (22.9)	6 (12.8)	7 (15.9)
60−69 yrs	32 (13.9)	6 (12.8)	6 (13.6)
70−79 yrs	8 (3.5)	3 (6.4)	3 (6.8)
≥80 yrs	3 (1.3)	1 (2.1)	1 (2.3)
Overall	231 (100)	47 (100)	44 (100)
			
All sites			
18−21 yrs	10 (3.6)	5 (8.3)	4 (7.5)
22−49 yrs	147 (52.9)	31 (51.7)	25 (47.2)
50−59 yrs	63 (22.7)	10 (16.7)	11 (20.8)
60−69 yrs	42 (15.1)	7 (11.7)	7 (13.2)
70−79 yrs	13 (4.7)	6 (10.0)	5 (9.4)
≥80 yrs	3 (1.1)	1 (1.7)	1 (1.9)
Overall	278 (100)	60 (100)	53 (100)

Discordant results were observed from 9 out of the 278 total specimens (Table S2). Eight specimens which were positive by the Lyra SARS-CoV-2 assay were negative by the Veritor SARS-CoV-2/Flu assay. Two of the eight specimens were associated with Ct values of <30; the other six had Ct values of ≥30 (Table S3). The BD MAX SARS-CoV-2 assay was used to resolve discordant results. Seven of the eight discordant specimens were positive by the BD MAX SARS-CoV-2 assay, while the remaining one was negative by the BD MAX SARS-CoV-2 assay. In addition, one specimen was positive by the Veritor SARS-CoV-2/Flu assay and negative by Lyra; this specimen was very close to the Veritor assay cutoff, and was negative by the BD MAX SARS-CoV-2 assay.

This clinical study was conducted at a time earlier than a regular flu season would occur. The mitigation approaches for the COVID-19 pandemic during 2020 also significantly reduced cases of viral respiratory diseases, including flu. Therefore, the concurrence of SARS-CoV-2 and influenza virus in this study was nonexistent. Only one flu A-positive and two flu B-positives were reported by the Veritor SARS-CoV-2/Flu test. One flu B-positive reported by Veritor was also shown as SARS-CoV-2 positive by both Veritor and Lyra reference results. These three specimens were further tested on Xpert Flu and resulted as negative, suggesting they were false-positives for flu A and flu B (Table S4).

Given the lack of prospective flu A and B samples in the clinical study and the identical antibody chemistry of Veritor Flu and Veritor SARS-CoV-2/Flu, remnant specimens were used to compare the sensitivity of the flu A and B detection of the Veritor SARS-CoV-2/Flu assay to that of Veritor Flu. These specimens included 75 retrospective, residual, de-identified, positive influenza specimens (40 flu A-positive and 35 flu B-positive) and 40 negative influenza A/B remnant specimens. The results from this testing were used to determine the PPA and NPA values of the two assays. For flu A detection, Veritor SARS-CoV-2/Flu had PPA and NPA values of 100% (95% CI: 91.2 to 100) and 100% (95.2 to 100), respectively; for Flu B, Veritor SARS-CoV-2/Flu had PPA and NPA values of 100% (95% CI: 90.0 to 100) and 100% (95.5 to 100), respectively (Table 3). Age stratifying the positive samples resulted in the ≥60-years-old group having the highest flu A-positivity ratio of all age groups (Table 4). However, most flu B-positive samples fell in the age group ranging from 6 to 59 years old.

**TABLE 3:** Performance of the Veritor SARS-CoV-2/Flu assay for detection of flu A and B compared that of the reference^*a*^

Characteristic	Flu A detection	Flu B detection
PPA, % (95% CI)	100 (91.2–100)	100 (90.0–100)
NPA, % (95% CI)	100 (95.2–100)	100 (95.5–100)
Veritor (+)/Ref (+), *n*	40	35
Veritor (+)/Ref (–), *n*	0	0
Veritor (–)/Ref (+), *n*	0	0
Veritor (–)/Ref (–), *n*	75	80
Kappa coefficient	1	1

a PPA, positive percent agreement; NPA, negative percent agreement. Reference method was the BD Veritor System Flu A+B assay.

**TABLE 4:** Influenza positivity distribution as determined by reference method or Veritor SARS-CoV-2/Flu across all age groups

Age group	*N* (%)	Reference method, *n* (%)		Veritor SARS-CoV-2/Flu, *n* (%)	
		Flu A	Flu B	Flu A	Flu B
≤5 yrs	15 (13.0)	3 (7.5)	8 (22.9)	3 (7.5)	8 (22.9)
6−21 yrs	23 (20.0)	6 (15)	13 (37.1)	6 (15)	13 (37.1)
22−59 yrs	39 (33.9)	11 (27.5)	13 (37.1)	11 (27.5)	13 (37.1)
≥60 yrs	38 (33.0)	20 (50.0)	1 (2.9)	20 (50.0)	1 (2.9)
Total	115 (100)	40	35	40	35

## DISCUSSION

The results presented here show PPA values for the Veritor SARS-CoV-2/Flu assay, compared to those of the Lyra SARS-CoV-2 assay, met FDA-EUA acceptance criteria for SARS-CoV-2 detection (86.7%; 95% CI: 75.8 to 93.1). Similarly, the Veritor SARS-CoV-2/Flu assay had a higher NPA value (99.5%; 95% CI: 97.4 to 99.9) compared to the same RT-PCR assay. PPA values from analyses, both excluding (91.5%) and including (86.7%) site D data, met FDA-EUA criteria for detection of SARS-CoV-2. Additionally, the flu detection portion of the Veritor SARS-CoV-2/Flu assay demonstrated 100% agreement with that of the FDA-cleared BD Veritor System Flu A+B assay. For flu A detection, the lower bound of the 95% CI was 91.2%; for flu B detection, the lower bound was 90.0%.

The Veritor SARS-CoV-2/Flu assay showed a reduced capacity to detect SARS-CoV-2 when the corresponding reference test result had Ct values of ≥30 during discordant testing. This is a common observation for antigen tests, since most of these assays rely on a threshold of target protein being available to flow by capillary action to initiate the antibody-antigen complex and detection reaction. Therefore, viable viral particles are required to produce threshold levels of antigen for detection (17). In contrast, PCR-based assays detect viral nucleic acid, which reflects both actively replicating virus and viral shedding. Viral load and analytical sensitivity of the reference RT-PCR assay may influence the sensitivity of the antigen test (17, 18). Thus, RT-PCR-based assays may seem more sensitive, but they do not necessarily reflect the infectivity of COVID-19, whereas antigen testing is a more specific approach for SARS-CoV-2 screening (compared to RT-PCR) and aligns with the infectiousness of the tested individual (19).

All 278 specimens tested for COVID-19 returned only one positive for flu A and two positives for flu B. These three specimens were further determined to be false-positives for flu A and B (Table S4). This could be due to a higher infection rate for SARS-CoV-2 or to the low activity of flu during the time of specimen collection (October 2020) (20). Because prospective collection for flu testing was not included with the specimen collection for SARS-CoV-2, de-identified remnants previously confirmed with influenza positivity by molecular methods were used. Since the antibody chemistry for detecting influenza virus is the same for both the FDA-cleared Veritor Flu assay and the Veritor SARS-CoV-2/Flu assay, the performance agreement of these two assays is sufficient to suggest that the Veritor SARS-CoV-2/Flu assay is equivalent to the Veritor Flu assay for detecting influenza. Indeed, the performance of Veritor SARS-CoV-2/Flu for detecting flu A and flu B showed 100% PPA with the reference method, Veritor Flu, suggesting the same sensitivity for influenza detection.

The 2020 to 2021 respiratory virus season concluded with an extremely low prevalence of influenza-like illness, likely due to the rigorous mitigation for the COVID-19 pandemic. However, in a season when the incidences of both COVID-19 and flu cases are high, the differential diagnosis of each agent for appropriate disease management could be made less challenging by using SARS-CoV-2/flu combination antigen tests. COVID-19 and influenza share similar transmission mechanisms and have overlapping clinical symptoms; however, the quarantine lengths and therapeutic approaches for each illness are not the same (21). While antiviral drugs, such as Tamiflu or Xofluza, are often given to influenza patients, remdesivir and corticosteroids are the primary medications that have been utilized to treat COVID-19 to date (8). After symptom onset, the minimum recommended quarantine period is 4 to 5 days for flu (22), but 10 days for COVID-19 (23). Therefore, accurate detection of both SARS-CoV-2 and flu A and B impacts not only the treatment plan, but also the quarantine period and the resulting loss of work and school attendance.

Different technologies are currently available for detecting SARS-CoV-2 and flu A/B viruses for the diagnosis of COVID-19 and flu, respectively (12, 17). Although the molecular-based approach is currently the laboratory method of choice due to its relatively high analytic and clinical sensitivity, rapid tests carry several advantages, including faster turnaround time and more straightforward implementation in decentralized health care settings for POC purposes (10, 12). Depending on the infrastructure and available resources in a healthcare facility, SARS-CoV-2/flu combination antigen tests could aid the process of distinguishing the detection and diagnosis of COVID-19 from flu for proper patient triage, disease mitigation, and treatment management.

In conclusion, the Veritor SARS-CoV-2/Flu assay met US FDA-EUA acceptance criteria for SARS-CoV-2 detection. The test sensitivity of the Veritor SARS-CoV-2/Flu assay for flu A and B detection agreed with that of the previously cleared Veritor System for Rapid Detection of or Veritor Flu for Flu A+B assay. Dual detection capability for the etiologic agents causing COVID-19 and influenza will be especially important for the duration of the COVID-19 pandemic as it overlaps with flu season, and could have a major impact in decentralized healthcare settings. This triplex assay will allow efficient differentiation between the two illnesses and support physicians regarding their diagnosis and thus, the proper treatment and disease management, for patients exhibiting similar symptoms.

## MATERIALS AND METHODS

### Specimens and assays.

This study was conducted as part of an FDA-EUA submission for the Veritor SARS-CoV-2/Flu test. Clinical performance of SARS-CoV-2 testing by the Veritor SARS-CoV-2/Flu assay was compared to that of a widely used EUA reference assay, the Lyra SARS-CoV-2 RT-PCR assay (“Lyra SARS-CoV-2”; Quidel, San Diego, CA, USA; limit of detection: 6,000 detectable units/mL). The BD MAX SARS-CoV-2 RT-PCR Assay (“MAX SARS-CoV-2”; Becton, Dickinson and Company; BD Life Sciences-Integrated Diagnostic Solutions, Sparks, MD, USA; limit of detection: 5,400 detectable units/mL) was used to resolve specimens with discrepant results between the Veritor SARS-CoV-2/Flu and Lyra SARS-CoV-2 tests. The Cepheid Xpert Xpress Flu/RSV (“Xpert Flu”; Cepheid, Sunnyvale, CA, USA) was used to resolve the positive flu results (potential discrepants) from the Veritor SARS-CoV-2/Flu assay during prospective SARS-CoV-2 testing. A method-comparison study for flu A and flu B testing was also conducted by comparing the data from Veritor SARS-CoV-2/Flu to that from Veritor Flu. Lyra testing was performed according to the manufacturer’s instructions for use at TriCore Reference Laboratories (Albuquerque, NM), while Veritor testing was performed at Becton, Dickinson and Company (BD Life Sciences-Integrated Diagnostic Solutions, San Diego, CA and Sparks, MD).

Subjects ≥18 years of age who were symptomatic for COVID-19 were enrolled within 7 DSO at six different sites across the U.S. to determine the clinical performance of the Veritor SARS-CoV-2/Flu for SARS-CoV-2 testing (Table S1 in the supplemental material). All specimens were collected by a healthcare provider. Two nasal swabs were collected in duplicate, with one swab rolled five times to collect mucus and cells in each nostril, and each swab then switched to the other nostril with the same rolling motion/duration. The swab designated for RT-PCR was placed in 3.0 mL of universal transport medium, and the swab designated for rapid antigen testing was stored dry in an empty container. Both swabs were stored at ≤–70°C or on dry ice within 30 minutes of collection for shipment (on dry ice) to testing sites. RT-PCR was conducted at a commercial laboratory and Veritor testing was conducted at a BD laboratory testing site in San Diego; all testing for RT-PCR and Veritor was performed in a blind manner with respect to results from the matched specimen. A total of 298 specimens were collected between 16 and 30 October 2020. Twenty specimens were excluded from the study for the following reasons: two were noncompliant specimens, and one was associated with an invalid reference (Lyra assay) result. The remaining seventeen (one noncompliant specimen and sixteen not tested) occurred at one site (site D), and were not included due to a collection procedure deviation (initiating freezing outside of the established 30 minutes window) that was communicated to the sponsor during study conduct (these sixteen specimens represented the last specimens collected by this site, and no additional data were collected from the site). The study used data from 278 subjects for the analysis. All study operators performing the Veritor SARS-CoV-2/Flu assay were blinded to reference method results.

The performance of the Veritor SARS-CoV-2/Flu for flu testing was evaluated by a method-comparison study using a separate set of retrospective specimens acquired from a repository. Whether the performance of the Veritor SARS-CoV-2/Flu for flu testing was comparable to that of Veritor Flu was determined. The influenza-positivity of these clinical remnants was predetermined using molecular tests at the reference laboratory, including 75 influenza-positive specimens (40 flu A-positive and 35 flu B-positive) and 40 influenza-negative specimens from subjects ranging from ≤5 to ≥60 years of age. The specimens were then de-identified and tested by the Veritor SARS-CoV-2/Flu and the Veritor Flu assays in a blind, randomized fashion. These residual specimens were nasopharyngeal (NP) swabs collected in universal viral transport (UVT) medium, which were obtained from qualified specimen vendors. The testing procedure for the Veritor SARS-CoV-2/Flu assay followed the protocol described for the Veritor Flu assay.

### Data analysis.

The primary outcome measures for this study were positive and negative percent agreement (PPA and NPA, respectively). Point estimates with 95% confidence interval [95% CI] were calculated using the Wilson score method for the Veritor SARS-CoV-2/Flu assay and compared to those for each reference method. The US FDA-EUA authorization acceptance criterion for test sensitivity for SARS-CoV-2 detection is a point estimate of ≥80% PPA compared that of the RT-PCR approach (24); for flu A and B, the criterion was a point estimate of ≥95% PPA with a lower bound of the 95% Confidence Interval of 85% when compared to the Veritor flu A+B Assay (13). Cohen’s kappa coefficient was applied to gauge agreement between the reference and index tests to classify results into mutually exclusive categories, according to the following formula: *k* = (*p*_o_ – *p*_e_)/(1 – *p*_e_), with values of <0, 0, and >0 indicating agreements worse than, no better or worse than, and better than that expected by chance. The data presented in this report met the criteria defined by FDA guidance for the Veritor SARS-CoV-2/Flu assay compared to reference assays. This article was prepared according to the Standards for Reporting of Diagnostic Accuracy Studies (STARD) guidelines for diagnostic accuracy studies reporting (25). Data will be made publicly available upon request.
