# Supplementary material for: Clinical Evaluation of BD Veritor SARS-CoV-2 and Flu A+B Assay for Point-Of-Care System
Source: Microbiol Spectr. 2022 Apr 12;10(2):e01807-21. doi: 10.1128/spectrum.01807-21 (PMC9045085; doi:10.1128/spectrum.01807-21)
Supplement: SUPPLEMENTAL FILE 1 — Supplemental material. Download spectrum.01807-21-s0001.pdf, PDF file, 0.2 MB [file spectrum.01807-21-s0001.pdf]

SUPPLEMENTAL INFORMATION
TABLE S1

Table S1. SARS-CoV-2 specimens collection sites.

Site	City	State	Enrollment
A	Palm Springs	FL	46
B	Savannah	GA	94
C	Ft. Lauderdale	FL	80
D[a]	Downers Grove	IL	64
E	Chattanooga	TN	11
F	St. Louis	MO	3
**TOTAL**			**298**

[a]The collection procedure was not consistent with the protocols utilized by other sites. The collection deviation was noted and therefore, the data was reported with and without this site.

TABLE S2

Table S2. List of discordant specimens.

Specimen ID	Veritor	Lyra	MAX	MAX Ct
1	-	+	+	N1: 29.06, N2: 29.48
2	-	+	+	N1: 26.38, N2: 29.48
3	-	+	-	N/A
4	-	+	+	N1: 30.79, N2: 32.72
5	-	+	+	N1: 30.43, N2: 32.38
6	-	+	+	N1: 38.27, N2: 32.71
7	-	+	+	N1: 31.82, N2: 33.95
8	-	+	+	N1: 30.86, N2: 321.40
9[a]	+	-	-	N/A

[a]Analysis of the Veritor Analyzer raw data demonstrated this specimen's results to be very close to the assay cut-off.

TABLE S3

Table S3. Comparison of Veritor SARS-CoV-2/Flu assay results with those from the Lyra SARS-CoV-2 assay, stratified by cycle threshold category, with and without D site.

**Without D Site**	**Lyra SARS-CoV-2**	
**Veritor SARS-CoV-2**	**Positive (Ct ≤ 30)**	**Positive (Ct > 30)**
**Positive**	42	1
**Negative**	0	4
**Total**	42	5
**PPA** (95% CI)	100% (91.6% - 100%)	20.0% (3.6% - 62.4%)

**All Sites**	**Lyra SARS-CoV-2**	
**Veritor SARS-CoV-2**	**Positive (Ct ≤ 30)**	**Positive (Ct > 30)**
**Positive**	50	2
**Negative**	2	6
**Total**	52	8
**PPA** (95% CI)	100% (92.6% - 100%)	100% (74.1% - 100%)

Abbreviations: Ct, PCR cycle threshold; PPA, positive percent agreement

TABLE S4

Table S4. List of influenza testing results from the prospective specimens using Veritor SARS-CoV-2/Flu and reference methods.

Specimen ID	Veritor (Flu A)	Veritor (Flu B)	Veritor (SARS-CoV-2)	Lyra (SARS-CoV-2)	Xpert (Flu)
1	+	-	-	-	-
2	-	+	-	-	-
3	-	+	+	+	-

FIGURE S1

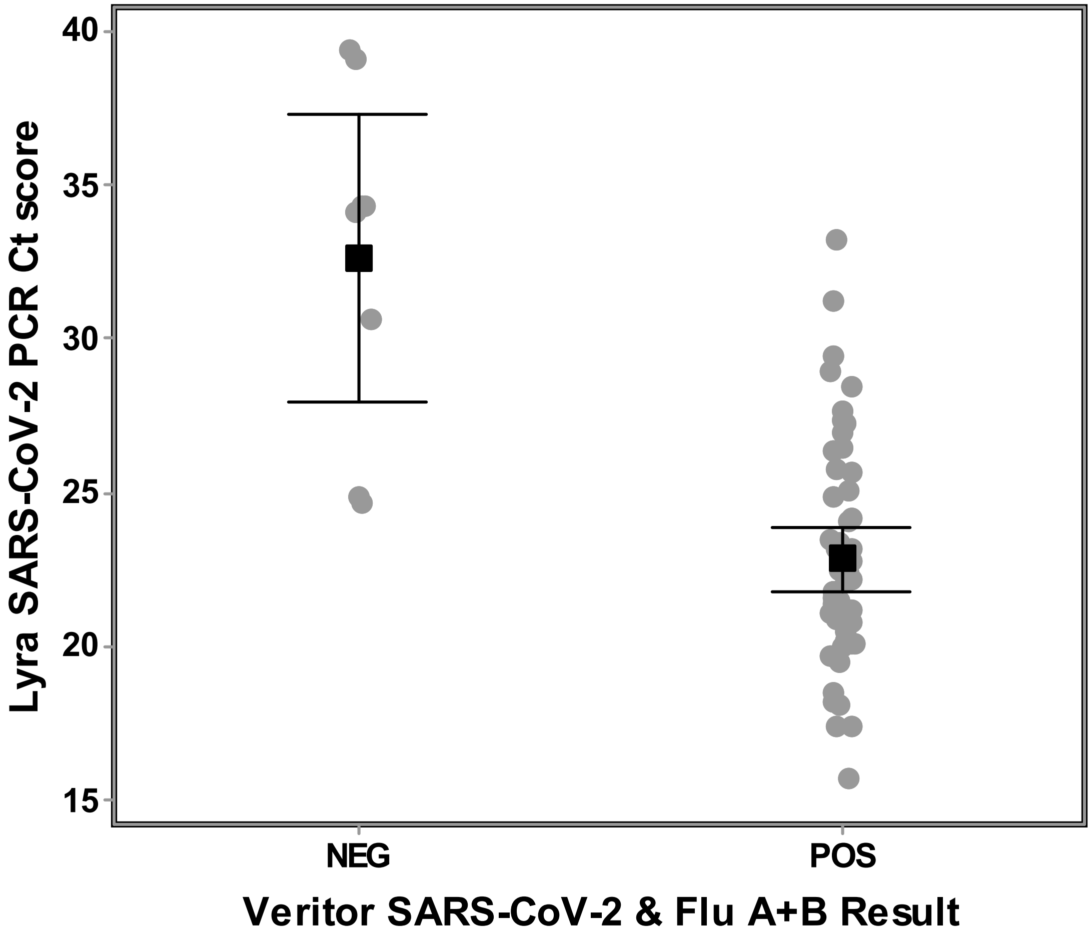

Figure S1. Individual plot of Lyra Ct score by Veritor SARS-CoV-2/Flu assay result. Individual negative and positive results for the Veritor SARS-CoV-2/Flu assay are plotted according to the Ct score from the Lyra SARS-CoV-2 PCR assay conducted on specimens obtained from the same individual. The grey circles indicate individual Veritor results. The black box indicates the mean for each group with 95% confidence intervals
